# Short stories from *Sphagnum* of rare species, taxonomy, and speciation

**DOI:** 10.1002/ece3.10356

**Published:** 2023-07-20

**Authors:** Magni Olsen Kyrkjeeide, Olena Meleshko, Kjell Ivar Flatberg, Kristian Hassel

**Affiliations:** ^1^ Norwegian Institute for Nature Research (NINA) Trondheim Norway; ^2^ Department of Natural History Norwegian University of Science and Technology Trondheim Norway

**Keywords:** genetic structure, microsatellites, molecular data, morphology, peatmosses, speciation, species identification

## Abstract

Conserving species and their genetic variation are a global priority to safeguard evolutionary potential in a rapidly changing world. Species are fundamental units in research and nature management, but taxonomic work is increasingly undermined. Increasing knowledge on the species genetic diversity would aid in prioritizing conservation efforts. *Sphagnum* is a diverse, well‐known bryophyte genus, which makes the genus suited to study speciation and cryptic variation. The species share specific characteristics and can be difficult to separate in the field. By combining molecular data with thorough morphological examination, new species have recently been discovered. Still, there are taxonomic uncertainties, even for species assessed on the IUCN Red List of threatened species. Here, we use molecular data to examine three rare species within the subgenus *Acutifolia* described based on morphological characters. All species have narrow distributions and limited dispersability. First, we confirm the genetic origin of *S. skyense*. Second, we show that *S. venustum* is a haploid species genetically distinct from morphologically similar species. Lastly, *S. nitidulum* was found to have a distinct haplotype, but cannot be genetically separated from other red *Acutifolia* species. We also found high genetic variation within red *Acutifolia* specimens, indicating the need of further morphological examination and possibly taxonomic revision. Until then, our results have shown that genetic data can aid in prioritizing targets of conservation efforts when taxonomy is unresolved. All three taxa should be further searched for by field biologists to increase knowledge about their distribution ranges.

## Graphical abstract

We used molecular data to resolve taxonomic uncertainty of three rare *Sphagnum* species described based on morhological characters. We confirm the parents of *Sphagnum skyense* and validate the species status of *S. venustum*. *Sphagnum nitidulum* has a distinct haplotype, but more data are needed to conclude whether it is a valid species.

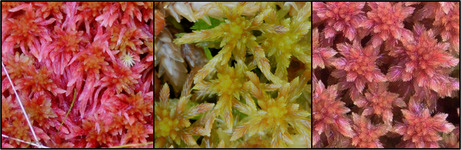

## 1 INTRODUCTION

Recognizing and describing species is an essential task in biology, as species are the fundamental biodiversity units used in many research fields and nature management. Correct species identification is crucial to, for instance, obtain species distribution maps and biodiversity estimates, which are commonly used in a wide range of ecological studies (Stropp et al., 2022). Also, conservation efforts are usually targeting species, and global, regional, and national Red lists make assessments at the species level. Furthermore, the inclusion of genetic data in conservation management and prioritizations is becoming increasingly recognized, as maintaining genetic diversity can make species more resilient to rapid environmental changes (Andrello et al., 2022). In fact, maintaining genetic diversity within species to safeguard their adaptive potential is listed under Goal A in the “Kunming‐Montreal Biodiversity Framework” (CBD, 2022). Thus, increasing knowledge about diversity among and within species is highly important to make informed decisions about where conservation efforts should be applied.

The genus *Sphagnum* L. is species‐rich and one of the most studied bryophyte genera, partly because it is the main driver of peat accumulation in boreal peatlands. Consequently, *Sphagnum* stores more carbon than any other plant genera (Yu et al., 2011). Evolutionary, the genus is on a long branch separated from the rest of the mosses, but with relatively recent, rapid diversification (Shaw et al., 2010). A major driver of speciation in *Sphagnum* seems to be adaptation to niches along the water table gradient within the mire landscape (Johnson et al., 2015; Rydin, 1986). Another important speciation mechanism is allopolyploidization, a process where two species hybridize and give viable offspring. Around 20% of *Sphagnum* species have originated as a result of allopolyploidization (Meleshko et al., 2018). Allopatric speciation due to barriers like the Atlantic Ocean is much less prevalent in bryophytes than seed plants, as Europe and North America share the majority of moss species (Carter et al., 2016; Frahm & Vitt, 1993). This is mainly explained by effective dispersal of bryophyte spores (Heinrichs et al., 2009; Kyrkjeeide, Hassel, Flatberg, Shaw, Brochmann, & Stenoien, 2016; Szövenyi et al., 2008). High species diversity of *Sphagnum* is found in the boreal region, a paradox considering the short time since the last glacial maximum. High dispersibility leading to colonization may explain this, but the role of geographical isolation in driving speciation in bryophytes is not well‐understood (but see Yousefi et al., 2019).

*Sphagnum* species are morphologically similar and often display phenotypic plasticity (Stenøien et al., 2014), and closely related species may have overlapping niches (Hassel et al., 2018; Yousefi et al., 2017). Thus, species delineation has been a debated topic within *Sphagnum* biology (Cronberg, 1989; Flatberg, 1986). Lately, investigating genetic variation has aided the identification of new species, even within common and well‐studied species such as *S. magellanicum* Brid. sensu lato (Hassel et al., 2018; Kyrkjeeide, Hassel, Flatberg, Shaw, Yousefi, & Stenoien, 2016; Shaw et al., 2022; Yousefi et al., 2019). Despite the effort to describe *Sphagnum* diversity, there are still unresolved taxonomical issues, even for species assessed in the IUCN Red List of threatened species (Gabriel & Sim‐Sim, 2019).

Taxonomic work is time‐consuming, and becoming a species expert may take years of training in the field and/or identification of microscopical characters in the laboratory. Field trips enable experts to collect specimens and enroll them in scientific collections. These collections have value for scientific discoveries and as a historical record of biodiversity (Pyke & Ehrlich, 2010; Suarez & Tsutsui, 2004), but not if specimens stay hidden in storage. For example, *S. beothuk* Andrus, described from Newfoundland, Canada (Andrus, 2006), was not detected in Europe before it was discovered to be genetically the same as specimens collected for years as a “dark morph” of *S. fuscum* (Schimp.) H. Klinggr. (Kyrkjeeide et al., 2015). Also, within the widespread *S. warnstorfii* Russow, extensively used in ecological studies (Bengtsson et al., 2016; Granath et al., 2010; Hájek et al., 2021), European specimens belonging to different genotypes seem to hold morphological variation (Mikulášková et al., 2015).

**FIGURE 1:**
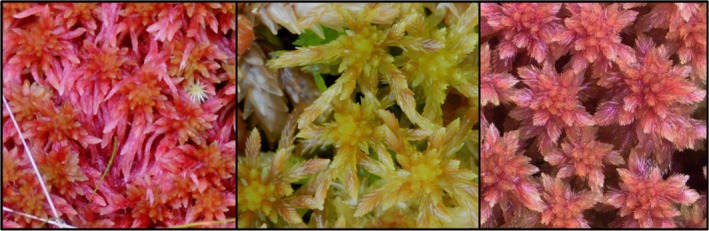
*Sphagnum skyense* (left), *S. venustum* (middle), and *S. nitidulum* (right) are three rare species described based on morphological data. Photos: K.I. Flatberg.

Here, we use molecular data to explore genetic differentiation and to clarify the taxonomic status of three rare species from *Sphagnum* subgenus *Acutifolia* (Wilson) A.J. Shaw: *S. skyense* Flatberg, *S. venustum* Flatberg, and *S. nitidulum* Warnstorf. All three species are described solely based on morphological data (Figure 1).

*Sphagnum skyense* was detected as a distinct red‐colored peat moss in the heathland on Isle of Skye, Scotland, and later described as a new species based on morphology (Flatberg, 1988a). The large size of *S. skyense* and its morphological resemblance to both *S. quinquefarium* (Lindb.) Warnst. and *S. subnitens* Russow & Warnst. lead to the hypothesis that this likely was an allopolyploid species. Genetic analysis including most species in subgenus *Acutifolia* (except *S. quinquefarium*) confirmed the allopolyploid origin, but suggested *S. warnstorfii* and *S. subnitens* as the likely progenitors of *S. skyense* (Shaw et al., 2005). No further attempt has been made in studying the ploidy level or the origin of *S. skyense*, with the latter remaining an open question for more than 30 years.

*Sphagnum venustum* is one of the most recent contributions of new species in *Acutifolia*. It was discovered and described based on collections from Labrador, eastern Canada (Flatberg, 2008), and later several new localities were reported from Quebec, Canada (Ayotte & Rochefort, 2019). In 2011, it was also discovered in Norway, but so far from one single site. Hill (2019) asked whether this single site occurrence of *S. venustum* could be a result of separate hybridization events on different continents. However, we hypothesis that it is a haploid species due to the small size, as allopolyploids tend to be larger than their progenitors (Flatberg, 1988a, 1988b).

*Sphagnum nitidulum* was described in 1911 and is still only known from one site on the Azorean island Terceira. It grows on soil in warm spots in the sulfurous fumaroles—a very rare substrate for *Sphagnum* species (Séneca & Söderström., 2009). The species has been seen as conspecific with *S. rubellum* (Andrews, 1941), but anyhow been included on several checklists (Gabriel & Sim‐Sim, 2019 and references therein). The type was assumed to be destructed, but it was photographed in 1991 (Gabriel & Sim‐Sim, 2019). Collections from the type locality in herbarium TRH were obtained in 2004 (four specimens) and 2021 (two specimens). *Sphagnum nitidulum* is one of the “red *Acutifolia* species.” Within this group, species delineation of *S. capillifolium* and *S. rubellum* has been debated and thoroughly studied, concluding that the two species may overlap morphologically (Cronberg, 1989), but are genetically distinct (Cronberg, 1996, 1998). Morphological overlap has also been observed between specimens identified as both *S. capillifolium* and *S. warnstorfii* (K. I. Flatberg, personal communication). Such specimens have been collected in mainland Europe the last two decades and tagged under different provisional names (specimens in TRH). We included these specimens (hereafter called conspecific specimens) to explore whether *S. nitidulum* is genetically distinct from other red *Acutifolia* species and whether the observed morphological similarities between *S. nitidulum* and deviating specimens of *S. warnstorfii* and *S. capillifolium* are reflected in molecular data.

We aim to identify the parental species of *S. skyense*, confirm the haploid level and the genetic distinctness of *S. venustum*, and evaluate whether *S. nitidulum* is genetically distinct from other “red *Acutifolia* specimens.”

## 2 MATERIALS AND METHODS

We used 72 *Sphagnum* specimens from the scientific collection (herbarium TRH) in Trondheim, Norway, collected in Europe and Eastern Canada. An overview of species and number of specimens are given in Table 1. DNA was extracted from the apical part of gametophytes, that is the middle part of the capitula, for each specimen using the NucleoSpin Plant II, Mini kit for plant DNA (Macherey‐Nagel) or E.Z.N.A.® HP Plant DNA Mini Kit (Omega Bio‐tek), following the manufacturers' protocols. The ploidy level of bryophytes is based on the gametophytic phase. Most *Sphagnum* species are haploid, whereas allopolyploid species have diploid or triploid gametophytes.

**TABLE 1:** Voucher information for *Sphagnum* specimens used in the molecular analyses.

DNA ID	TRH ID	Herb. assigned taxon	Country	Province/county	Longitude	Latitude	Year	Collector	Assigned taxon in analyses
**ss1**	728,102	*S. skyense*	Scotland	Highland			1987	KIF	Skyense
**ss3**	728,099	*S. skyense*	Scotland	Highland			2004	MOH	Skyense
**ss4**	741,748	*S*. cf. *skyense*	Ireland	Tipperary			2005	NGH	Skyense
**ss5**	93,374	*S. subnitens* ssp*. subnitens*	Norway	Vestland	4,97883	60,59546	2017	MJ, CP, MOK, KIF	Subnitens
**ss6**	93,414	*S. subnitens* ssp*. subnitens*	Norway	Vestland	5,10753	59,99331	2017	MJ, CP, MOK, KIF	Subnitens
**ss7**	728,280	*S. subnitens* ssp*. subnitens*	Scotland	Highland			1987	KIF	Subnitens
**ss8**	120,164	*S. subnitens* ssp*. subnitens*	Ireland	Mayo	−9,51535	54,10317	2011	MOK, KOK	Subnitens
**ss9**	728,298	*S. subnitens* ssp*. subnitens*	Wales	Gwynedd			1980	KIF	Subnitens
**ss10**	93,420	*S. quinquefarium*	Norway	Vestland	5,20637	59,59257	2017	MJ, CP, MOK, KIF	Quinquefarium
**ss11**	93,405	*S. quinquefarium*	Norway	Vestland	5,10615	59,99316	2017	MJ, CP, MOK, KIF	Quinquefarium
**ss12**	120,123	*S. quinquefarium*	Scotland	Highland	−6,06299	57,17806	2011	MOK	Quinquefarium
**ss13**	4802	*S. quinquefarium*	Scotland	Scottish Borders	−2,85016	55,19173	2014	KH	Quinquefarium
**ss68**	109,503	*S. quinquefarium*	Scotland	Highland	−6,24462	57,42046	2019	NGH	Quinquefarium
ss23	93,432	*S. capillifolium*	Norway	Vestland	5,19754	59,61486	2017	MJ, CP, MOK, KIF	Capillifolium
ss24	673,473	*S. capillifolium*	Scotland	Highland			1987	KIF	Capillifolium
ss26	13,176	*S. capillifolium*	Scotland	Highland	−5,01957	58,19938	2015	KH	Capillifolium
ss28	94,003	*S. capillifolium*	Austria	Salzburg	13,78167	47,08359	2017	KIF, CS	Capillifolium
ss29	38,148	*S. capillifolium*	Slovakia	Prešov Region	20,22992	49,2195	2016	KH	Capillifolium
ss30	98,303	*S. capillifolium*	Slovakia	Prešov Region	20,22212	49,21094	2016	KIF	Capillifolium
ss31	93,386	*S. capillifolium*	Norway	Vestland	5,01412	60,56181	2017	MJ, CP, MOK, KIF	Capillifolium
ss36	93,415	*S. capillifolium*	Norway	Vestland	5,10753	59,99331	2017	MJ, CP, MOK, KIF	Capillifolium
ss37	93,414	*S. capillifolium*	Norway	Vestland	5,10753	59,99331	2017	MJ, CP, MOK, KIF	Capillifolium
ss39	740,734	*S. capillifolium*	Norway	Trøndelag	10,41161	63,20311	2003	KIF	Capillifolium
224	108,510	*S. rubellum*	Norway	Trøndelag	11,21917	64,23135	2019	KH, OM, MOK, KIF	Conspecific
230	115,591	*S*. cf. *capillifolium* × *warnstorfii*	Norway	Trøndelag	9,099786	63,06362	2016	KIF	Conspecific
241	148,557	*S*. cf. *capillifolium* × *warnstorfii*	Norway	Trøndelag	10,46162	63,38908	2019	KIF	Conspecific
ss21	93,450	*S*. cf. *warnstorfii*	Norway	Vestland	5,19795	59,61641	2017	MJ, CP, MOK, KIF	Conspecific
ss27	94,018	*S. capillifolium*	Austria	Salzburg	13,67968	47,08395	2017	KIF, CS	Conspecific
ss34	94,041	*S*. cf. *capillifolium* × *warnstorfii*	Austria	Oberosterreich	12,95769	48,0541	2017	KIF	Conspecific
ss35	94,020	*S*. cf. *capillifolium* × *warnstorfii*	Austria	Salzburg	13,67906	47,08405	2017	KIF, CS	Conspecific
SW01	148,553	*S*. cf. *warnstorfii*	Denmark	Jylland	9,135816	56,37582	2021	IG	Conspecific
SN11	727,588	*S*. cf. *nitidulum*	Portugal	Azores			2004	KIF	Nitidulum
SN12*	121,476	*S. nitidulum*	Portugal	Azores	−27,2	38,73333	2021	EFC	Nitidulum
SN13*	121,475	*S. nitidulum*	Portugal	Azores	−27,2167	38,73333	2021	EFC	Nitidulum
SN14*	727,587	*S*. cf. *nitidulum*	Portugal	Azores			2004	KIF	Nitidulum
RB01**	728,039	*S*. cf. *rubellum*	Portugal	Azores			2004	KIF	Nitidulum
221	725,290	*S. rubellum*	Canada	Quebec	−57,1541	51,43078	2007	BF, KIF	Rubellum
222	725,305	*S. rubellum*	Canada	Newfoundland and Labrador	−56,7302	51,57122	2007	BF, KIF	Rubellum
223	37,834	*S. rubellum*	Norway	Trøndelag	11,43851	64,70585	2016	KH	Rubellum
240	148,556	*S. rubellum*	Norway	Trøndelag	10,46162	63,38908	2019	KIF	Rubellum
242	148,558	*S. rubellum*	Norway	Trøndelag	10,46162	63,38908	2019	KIF	Rubellum
ss15	11,185	*S*. cf. *warnstorfii*	Norway	Vestland	5,16446	59,65667	2015	KH	Rubellum
ss25	120,124	*S. rubellum*	Scotland	Highland	−6,37338	57,37548	2011	MOK	Rubellum
ss32	98,281	*S*. cf. *capillifolium*	Czech Republic	Pardubický kraj	15,96429	49,73827	2016	KIF	Rubellum
ss33*	98,279	*S*. cf. *capillifolium*	Czech Republic	Pardubický kraj	15,96429	49,73827	2016	KIF	Rubellum
ss61	158,861	*S. rubellum*	Norway	Trøndelag	10,06233	63,97804	2002	KIF	Rubellum
ss63	725,288	*S. rubellum*	Canada	British Columbia	−126	49,16667	2000	KIF	Rubellum
229	115,591	*S. warnstorfii*	Norway	Trøndelag	9,099786	63,06362	2016	KIF	Warnstorfii
**ss14**	728,537	*S. warnstorfii*	Scotland	Highland			1987	KIF	Warnstorfii
**ss16**	740,618	*S. warnstorfii*	Norway	Vestland	5,64284	60,41393	2008	KIF	Warnstorfii
ss17	38,119	*S. warnstorfii*	Czech Republic	Pardubický kraj	15,96413	49,73867	2016	KH	Warnstorfii
ss18	98,276	*S. warnstorfii*	Czech Republic	Pardubický kraj	15,93139	49,70528	2016	KIF	Warnstorfii
ss19	93,992	*S. warnstorfii*	Austria	Salzburg	13,86189	47,18654	2017	KIF, CS	Warnstorfii
ss20	94,022	*S. warnstorfii*	Austria	Salzburg	13,7101	47,10077	2017	KIF, CS	Warnstorfii
**ss65**	109,505	*S. warnstorfii*	Scotland	Highland	−6,22782	57,4338	2019	NGH	Warnstorfii
**ss66**	109,504	*S. warnstorfii*	Scotland	Highland	−6,22782	57,4338	2019	NGH	Warnstorfii
ss67	109,506	*S. warnstorfii*	Scotland	Highland	−6,22782	57,4338	2019	NGH	Warnstorfii
ss64	728,492	*S. venustum*	Canada	Newfoundland and Labrador	−56,3042	51,97892	2007	BF, KIF	Venustum
ss70	741,059	*S. venustum*	Norway	Trøndelag	11,69203	63,93091	2011	KIF	Venustum
ss71	728,496	*S. venustum*	Canada	Quebec	−72,6333	53,85	2010	MW	Venustum
220	741,060	*S. venustum*	Norway	Trøndelag	11,69203	63,93091	2011	KIF	Venustum
217	724,524	*S. beothuk*	Canada	Newfoundland and Labrador	−56,7053	51,62902	2007	BF, KIF	Beothuk
213	108,518	*S. beothuk*	Norway	Trøndelag	11,24113	64,57635	2019	KH, OM, MOK, KIF	Beothuk
214	108,530	*S. beothuk*	Norway	Trøndelag	11,07712	64,4292	2019	KH, OM, MOK, KIF	Beothuk
215	724,518	*S. beothuk*	Canada	Newfoundland and Labrador	−55,6243	52,37197	2007	BF, KIF	Beothuk
219	115,819	*S. fuscum*	Canada	Quebec	−72,2329	52,81405	2018	LB, MFI	Fuscum
210	724,520	*S. fuscum*	Canada	Quebec	−57,2362	51,50422	2007	BF, KIF	Fuscum
212	724,523	*S. fuscum*	Canada	Newfoundland and Labrador	−56,7088	51,66303	2007	BF, KIF	Fuscum
209	114,017	*S. fuscum*	Norway	Trøndelag	11,21456	64,26283	2020	KH, KIF, OM	Fuscum
211	676,317	*S. fuscum*	Norway	Trøndelag	11,6934	63,93074	2013	TP	Fuscum
ss58	158,973	*S. fuscum*	Norway	Trøndelag	10,51795	64,09067	2002	KIF	Fuscum
ss60	724,509	*S. fuscum*	Canada	British Columbia			2000	KIF	Fuscum

*Note*: The specimens were analyzed in three different datasets: the *Actuifolia* dataset included all specimens except *S. skyense* and samples marked with * (assumed to be clones sampled at the same site), the ‘red *Acutifolia*’ dataset further excluded samples marked as ** (identical haplotypes), and the *S. skyense* dataset that included all individuals in bold. The specimen order in the table is the same as the specimen order in the boxplots in Figures 2 and 3 from left to right. Collectors: BF, B. Flatberg; CP, C. Pötsch; CS, C. Schröck; EF, E. Feldmayer‐Christe; IG, I. Goldberg; KH, K. Hassel; KIF, K.I. Flatberg; KOK, K.O. Kyrkjeeide; LB, L. Bourgouin; MFI, M.‐F. Indorf; MJ, M. Jokerud; MOH, M.O. Hill; MOK, M.O. Kyrkjeeide; MW, M. White; NGH, N.G. Hodgetts; OM, O. Meleshko; TP, T. Prestø.

We used microsatellite markers to explore genetic diversity of the specimens. These markers have been frequently used as a mean of identifying new species (e.g., Karlin et al., 2008) and genetic structuring patterns (Kyrkjeeide, Hassel, Flatberg, Shaw, Yousefi, & Stenoien, 2016), and they allow identification of ploidy levels as allopolyploid species have more than one allele present at several loci (Kyrkjeeide et al., 2019; Ricca et al., 2008). Furthermore, patterns obtained by microsatellite data has been confirmed using much larger SNP datasets (Duffy et al., 2020; Shaw et al., 2022). Altogether, 15 microsatellite markers (1, 7, 9, 12, 17, 19, 20, 22, 29, 30, 56, 65, 68, 78, 93) were amplified for all specimens and genotyped in GeneMapper (Table 2, see e.g., Kyrkjeeide, Hassel, Flatberg, Shaw, Brochmann, & Stenoien, 2016 for methods).

**TABLE 2:** Allele data of *Acutifolia* specimens from 15 microsatellite markers (ssr) developed for *Sphagnum*.

DNA ID	Assigned name in analyses	ssr1	ssr7	ssr12	ssr68	ssr19	ssr93	ssr29	ssr30	ssr9	ssr56	ssr20	ssr22	ssr17	ssr65	ssr78
**ss1**	*skyense*	245	189/198	117	218/225	264	221/253	198	127/139	187/189	200/206	277/282	94/103	158/0	190	201/213
**ss3**	*skyense*	245	189/198	117	218/225	264	221/253	198	127/139	187/189	200/206	277/282	94/103	158/164	190	201/213
**ss4**	*skyense*	245	189/200	117	218/225	264	221/250	198	127/139	187/187	200/206	277/282	94/103	158/164	190	201/207
**ss5**	*subnitens*	245	189	117	225	264	221	198	127	187	209	282	94	164	190	207
**ss6**	*subnitens*	245	189	117	225	264	221	198	127	187	203	282	94	164	190	207
**ss7**	*subnitens*	245	189	117	225	264	221	198	127	187	203	282	94	164	190	207
**ss8**	*subnitens*	245	197	117	225	264	221	198	127	187	197	282	94	164	190	213
**ss9**	*subnitens*	245	189	117	225	264	221	198	127	187	197	282	94	164	190	207
**ss10**	*quinquefarium*	245	198	117	218	256	0	201	139	186	206	277	103	158	190	201
**ss11**	*quinquefarium*	245	198	117	218	256	250	198	139	189	206	277	103	156	190	201
**ss12**	*quinquefarium*	245	198	117	218	256	253	201	139	189	206	277	103	156	190	201
**ss13**	*quinquefarium*	245	198	117	218	256	0	198	139	186	206	277	103	158	190	201
**ss68**	*quinquefarium*	245	198	117	218	256	253	198	139	186	206	277	103	156	190	201
ss23	*capillifolium*	245	177	119	222	256	229	201	137	172	239	276	106	155	187	195
ss24	*capillifolium*	245	179	119	222	256	229	201	137	185	239	276	106	161	187	213
ss26	*capillifolium*	245	177	119	222	270	229	201	137	185	230	276	106	161	187	195
ss28	*capillifolium*	245	177	119	222	256	229	202	137	185	221	277	106	161	190	195
ss29	*capillifolium*	245	177	119	222	256	229	201	137	172	233	276	106	155	187	213
ss30	*capillifolium*	245	177	119	222	256	229	200	137	168	233	277	106	155	187	195
ss31	*capillifolium*	245	177	119	222	256	229	201	137	185	221	276	106	161	187	195
ss36	*capillifolium*	245	177	119	222	256	229	200	137	169	230	276	106	161	187	195
ss37**	*capillifolium*	245	177	119	222	256	229	200	137	169	230	276	106	161	187	195
ss39	*capillifolium*	245	177	119	222	256	229	201	137	172	239	277	106	155	187	195
224	*conspecific*	245	177	119	225	258	229	198	137	185	215	274	109	155	184	210
230	*conspecific*	245	179	119	225	267	247	204	137	0	215	274	109	155	185	195
241	*conspecific*	245	186	123	223	256	229	198	137	184	215	274	112	157	190	210
ss21	*conspecific*	245	177	123	225	256	229	195	143	169	215	274	109	157	190	195
ss27**	*conspecific*	245	0	119	223	264	229	198	137	162	224	274	112	157	184	195
ss34	*conspecific*	245	179	123	225	0	229	195	137	184	215	274	109	155	190	207
ss35	*conspecific*	245	0	119	223	264	229	198	137	162	224	274	112	157	184	195
SW01	*conspecific*	245	179	119	225	267	229	195	137	184	215	300	109	152	184	207
SN1*3*	*nitidulum*	245	175	119	231	259	247	204	147	169	233	275	109	157	190	201
SN11	*nitidulum*	245	175	119	231	259	247	204	147	169	233	275	109	157	190	201
SN12*	*nitidulum*	245	175	119	231	259	247	204	147	169	233	275	109	157	190	201
SN14*	*nitidulum*	245	175	119	231	259	247	204	147	169	233	275	109	157	190	201
RB01**	*nitidulum*	245	175	119	231	259	247	204	147	169	233	275	109	157	190	201
221	*rubellum*	245	177	119	231	264	232	198	137	195	221	277	112	155	0	216
222	*rubellum*	245	173	119	225	259	247	198	137	180	227	295	109	155	184	210
223	*rubellum*	245	0	119	225	264	241	195	137	0	230	295	112	157	196	207
240	*rubellum*	245	187	125	225	264	229	196	137	169	191	274	94	157	194	201
242	*rubellum*	245	179	125	225	263	241	200	147	179	224	298	109	157	190	210
**ss15**	*rubellum*	245	177	123	225	259	229	204	139	169	233	295	109	152	184	210
ss25	*rubellum*	245	179	119	225	266	244	201	143	169	224	274	106	152	184	204
ss32	*rubellum*	245	179	123	228	264	244	196	137	169	224	295	109	157	190	195
ss33*	*rubellum*	245	179	123	228	264	244	196	137	169	224	295	109	157	190	195
ss61	*rubellum*	245	173	125	225	264	232	204	137	169	224	298	90	157	196	195
ss63	*rubellum*	245	181	123	225	274	232	196	147	172	218	274	106	152	194	201
229	*warnstorfii*	245	190	119	225	267	244	201	139	172	194	290	100	155	187	204
**ss14**	*warnstorfii*	245	194	119	225	270	244	201	141	172	188	289	100	155	181	204
**ss16**	*warnstorfii*	245	190	0	225	267	247	201	139	172	212	289	100	157	187	0
ss17	*warnstorfii*	245	190	121	225	267	247	201	141	172	224	289	100	155	187	195
ss18	*warnstorfii*	245	190	0	225	270	244	201	139	172	212	289	100	155	187	204
ss19	*warnstorfii*	245	190	117	225	267	243	201	139	179	212	292	103	155	187	204
ss20	*warnstorfii*	245	190	0	225	270	243	201	139	179	194	289	103	155	187	0
ss65**	*warnstorfii*	245	190	119	0	269	244	201	141	196	221	289	103	155	181	0
**ss66**	*warnstorfii*	245	190	119	225	265	247	200	141	178	224	289	100	155	187	0
ss67	*warnstorfii*	245	190	119	225	269	244	201	141	196	221	289	103	155	181	201
220	*venustum*	245	175	119	218	282	251	198	137	196	227	277	106	155	191	204
ss64	*venustum*	245	175	119	218	279	251	201	147	196	227	277	106	155	191	195
ss70**	*venustum*	245	175	119	218	282	251	0	137	196	227	277	106	155	191	204
ss71	*venustum*	245	175	119	218	273	251	195	143	196	227	277	106	155	191	204
217	*beothuk*	245	179	121	218	256	250	198	127	182	206	277	106	0	194	201
213	*beothuk*	245	179	121	218	256	238	198	127	182	206	277	106	157	194	201
214	*beothuk*	245	179	121	218	256	238	198	127	182	206	277	106	157	194	201
215	*beothuk*	245	179	121	218	0	247	198	127	182	206	277	106	157	194	201
219	*fuscum*	245	189	121	225	256	241	198	127	182	209	294	103	158	175	198
210	*fuscum*	245	189	121	225	256	241	198	130	186	212	288	103	158	176	198
212	*fuscum*	245	189	121	225	256	241	198	127	182	209	288	109	158	176	198
209	*fuscum*	245	193	121	225	256	241	198	130	182	212	288	97	158	176	198
211	*fuscum*	245	193	121	227	256	241	198	130	186	212	291	103	158	176	198
ss58	*fuscum*	245	189	121	225	256	241	198	130	186	215	291	106	158	176	198
ss60	*fuscum*	245	189	121	225	255	241	198	127	186	212	291	103	153	176	198

*Note*: The DNA ID correspond to the ID in Table 1 and Figure 4. Specimens were analyzed in three different datasets (see main text and Table 1). The specimen order in the table is the same as the specimen order in the boxplots in Figures 2 and 3 from left to right. *Sphagnum skyense* named skyense under Assigned name in analyses, is diploid and have two alleles at each microsatellite.

After visually studying the number of alleles identified for each morphological species, three datasets were prepared for statistical analyses. The first dataset included all specimens identified as haploid (one allele at each locus), this only excluded *S. skyense*. In addition, four samples were excluded as they were collected at the same site and had identical haplotypes, and hence were likely clones (see Table 2). A total of 65 specimens were included in the dataset and we hereafter call it the *Acutifolia* dataset. The second dataset, hereafter called the “red *Acutifolia*” dataset, included specimens of *S. rubellum*, *S. capillifolium*, *S. warnstorfii*, *S. nitidulum* and conspecific specimens, and also *S. venustum*, as analyses of the *Acutifolia* dataset show that this species cluster with *“*red *Acutifolia*” (see below). Specimens with identical haplotypes were removed from the dataset, and a total of 39 specimens were analyzed. The third dataset, hereafter called the *S. skyense* dataset, included *S. skyense* and the hypothesized parental species: *S. subnitens*, *S. quinqefarium*, and *S. warnstorfii*. In total, 17 specimens were included in this dataset and scored as diploid individuals, with the second allele scored as missing for the three latter species.

The software Structure (Falush et al., 2003, 2007; Pritchard et al., 2000) was used to identify the number of genetic clusters and potential admixture among individuals of morphologically described taxa (sensu van Hengstum et al., 2012). The analysis was run for the first dataset with the number of genetic clusters (*K*) ranging from 1 to 10, to explore clustering among all *Acutifolia* species. The second dataset was run using *K* = 2–6. The dataset included five morphologically described species and seven specimens that were collected as conspecific (Table 1). The third dataset was run for *K* = 3, as *S. skyense* is described as a hybrid species and the aim of the analyses were to detect potential admixture between the parental species. For each *K*, the analyses were replicated 10 times with a burnin of 100,000 steps followed by a Monte Carlo Markov chain of 200,000 steps. We ran Structure with different settings for the first dataset applying the admixture model and both the correlated and independent allele frequencies model. The correlated allele frequencies model is better at detecting distinct populations that are closely related (Porras‐Hurtado et al., 2013), and this model was used when analyzing the second and third datasets. The results were further assessed using Clumpak (Kopelman et al., 2015) and visualized with StructureSelector (Li & Liu, 2017).

The data were also explored in GenAlEx v6.501 (Peakall & Smouse, 2012). A genetic distance matrix was made of the *Acutifolia* dataset and the “red Acutifolia” dataset using the haploid (ssr) option and interpolating missing data. The distance matrices were used in PCoA analyses with the covariance‐standardized method in GenAlEx, but for the *Acutifolia* dataset also in SplitsTree v4.19.0 (Huson & Bryant, 2006) to make a Neighbor‐Net network (Bryant & Moulton, 2002). A PCoA was also made for the *S. skyense* dataset, but the distance matrix was made with the codominant option and the haploid specimens scored as homozygotes to avoid missing data at the second allele.

## 3 RESULTS

**FIGURE 2:**
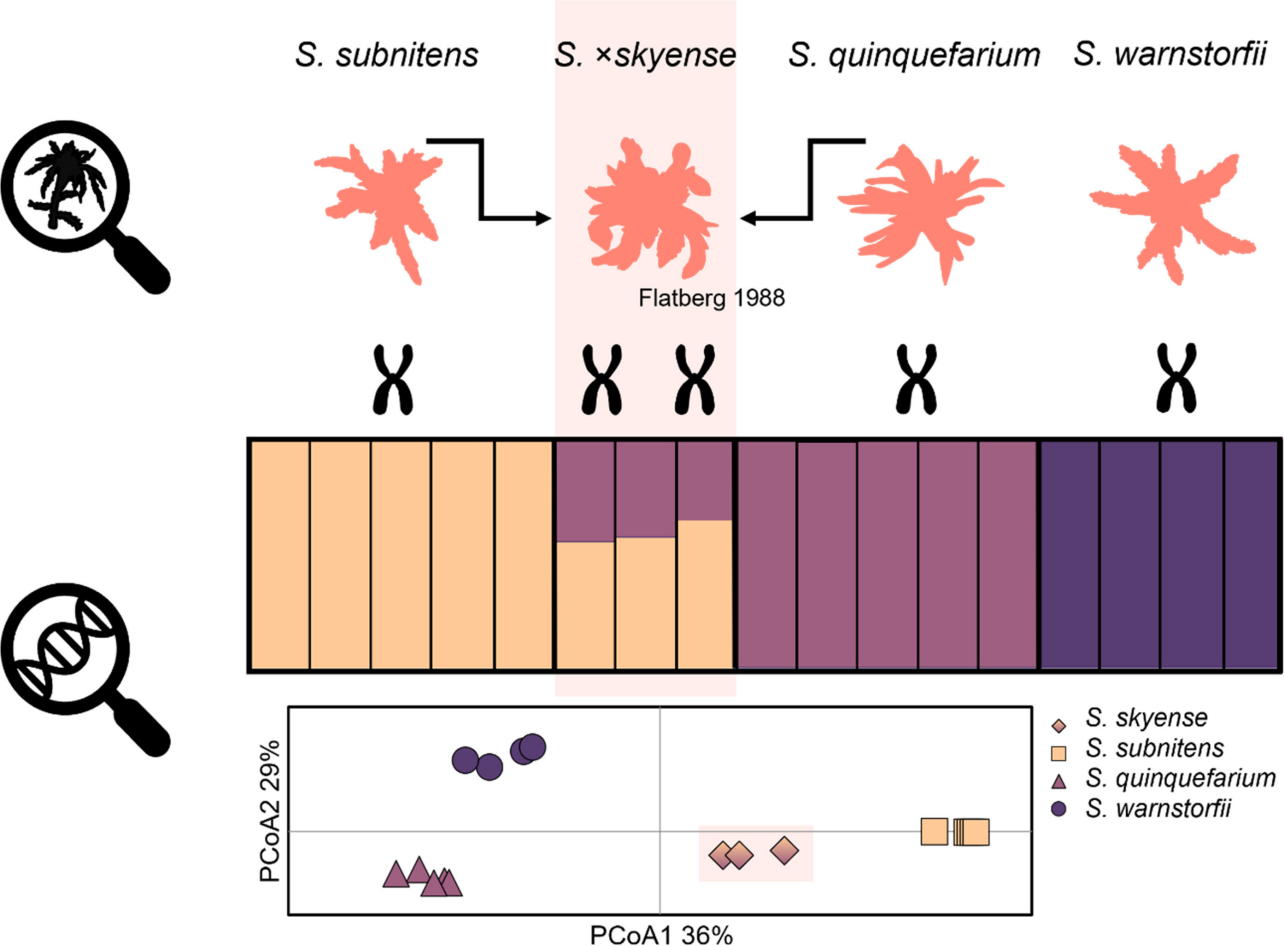
Microsatellite data of *Sphagnum* × *skyense* show that the species has two alleles at most of its loci, strongly indicating that it is a diploid species originating from hybridization. The barplot shows the result of STRUCTURE analysis of microsatellite data of *S*. × *skyense* and three hypothesized parental species. The result confirms that *S*. × *skyense* is an admixed species between *S. subnitens* and *S. quinquefarium*. This is also evident in the PCoA (below) which shows that *S*. × *skyense* is placed between its parental species. In the barplot, each bar represents one individual. The order of the bars, from left to right, follows the order of individuals listed in Table 1 (dataset *S*. × *skyense*).

We used the number of alleles recognized for each locus to confirm the ploidy level of *S. skyense* and *S. venustum* (sensu Ricca et al., 2008). Our results confirm that *S. venustum* is a haploid species, as only one allele was identified for each locus, while *S. skyense* is diploid as 10 of 15 loci have two alleles. At the five loci with only one allele, four of them have the same allele in *S. skyense*, *S. subnitens* and *S. quinquefarium* (Table 2). Furthermore, a visual comparison of alleles across the *S. skyense* dataset, shows that the alleles in the heterozygote loci are mainly the same as alleles found in *S. subnitens* and *S. quinquefarium*. The Structure analysis of this dataset shows that *S. skyense* is indeed admixed between *S. subnitens* and *S. quinquefarium*, strongly supporting that they are the parental species of *S. skyense* (Figure 2).

Also, specimens of *S. venustum* had one allele at each loci, thus, the species is haploid. A sample collected as *S*. cf. *rubellum* in the Azores (Terceira) was genetically identical to the samples collected as *S. nitidulum* (Table 2). The Azores specimens share alleles with other *Acutifolia* species, but the same alleles overlap at maximum four loci with other specimens (Table 2). All the conspecific specimens had one allele at each locus; hence, they were all haploids, even though many of them were morphologically assigned as hybrids (Table 1).

**FIGURE 3:**
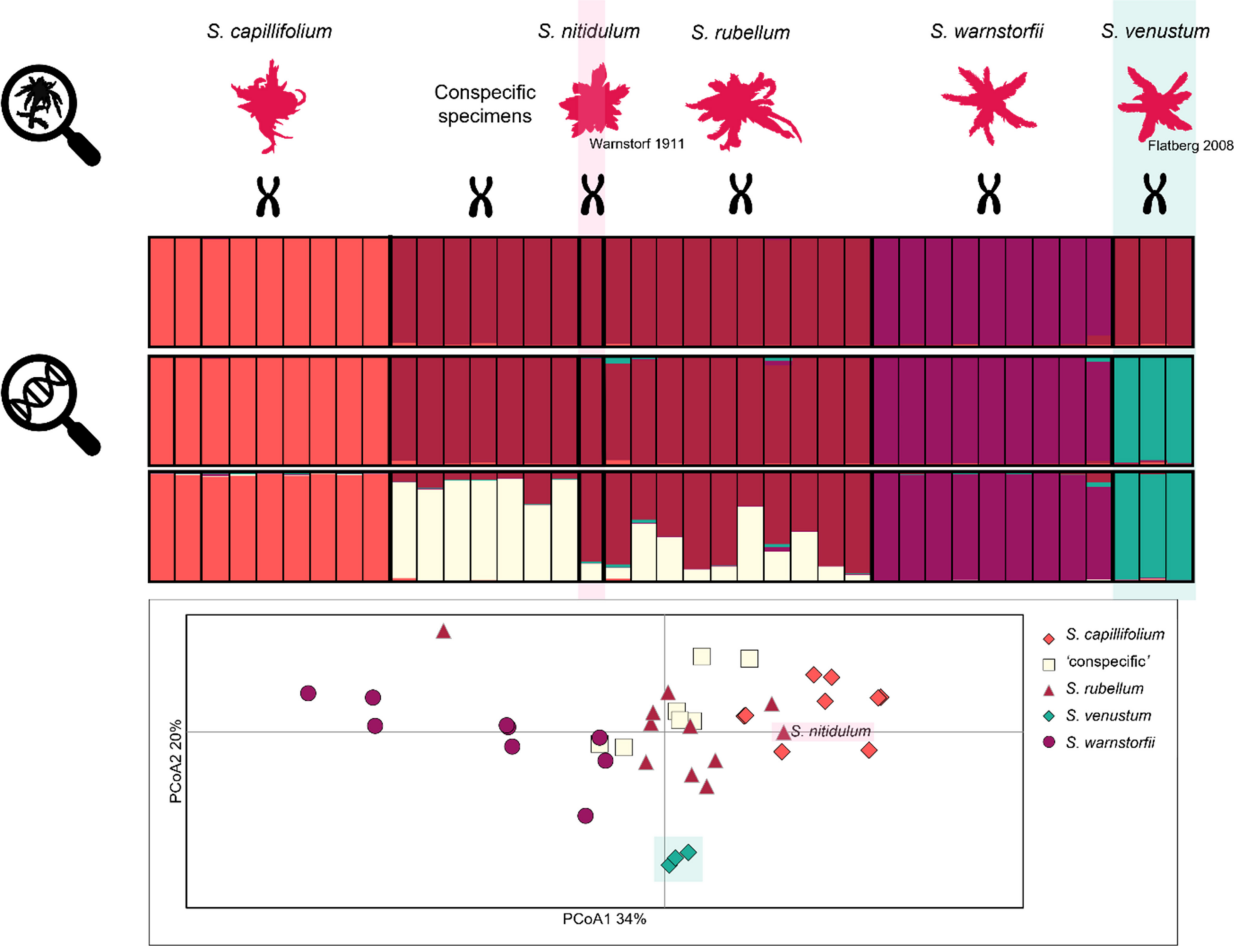
Barplots show the result of STRUCTURE analysis of 15 microsatellite data of five morphologically described *Sphagnum* sect. *Acutifolia* species and seven specimens assigned as conspecific. The order of the bars, from left to right, follows the order of individuals listed in Tables 1 and 2. The barplots show the results for different number of genetic clusters: 3 (results of 10/10 runs), 4 (results of 8/10) and 5 (10/10 runs). The PCoA (below) shows that *S. venustum* is separated from the other species, that are overlapping. The *S. nitidulum* sample (named and colored pink in the plot) was included as *S. rubellum* in the analysis and found within specimens of *S. rubellum* and *S. capillifolium*.

The Structure analysis of the *Acutifolia* dataset shows that *S. venustum* mainly clusters with “red *Acutifolia*” species and that morphological species separate into different genetic clusters as the value of *K* increases. The Azorean specimens cluster with *S. rubellum* at *K* = 2–5, but as a separate cluster at *K* = 7–10. However, in analyses of the “red *Acutifolia*” dataset, where only unique haplotypes were included, the Azorean specimen cluster with *S. rubellum* at all *K*‐values (Figure 3). Furthermore, this analysis separates *S. capillifolium*, *S. warnstorfii*, *S. rubellum* and “conspecific” specimens, and *S. venustum* into different genetic clusters at *K* = 4, while the “conspecific” specimens form a fifth genetic cluster at *K* = 5 (Figure 3). Specimens of *S. rubellum* show varying extent of admixture with this cluster.

**FIGURE 4:**
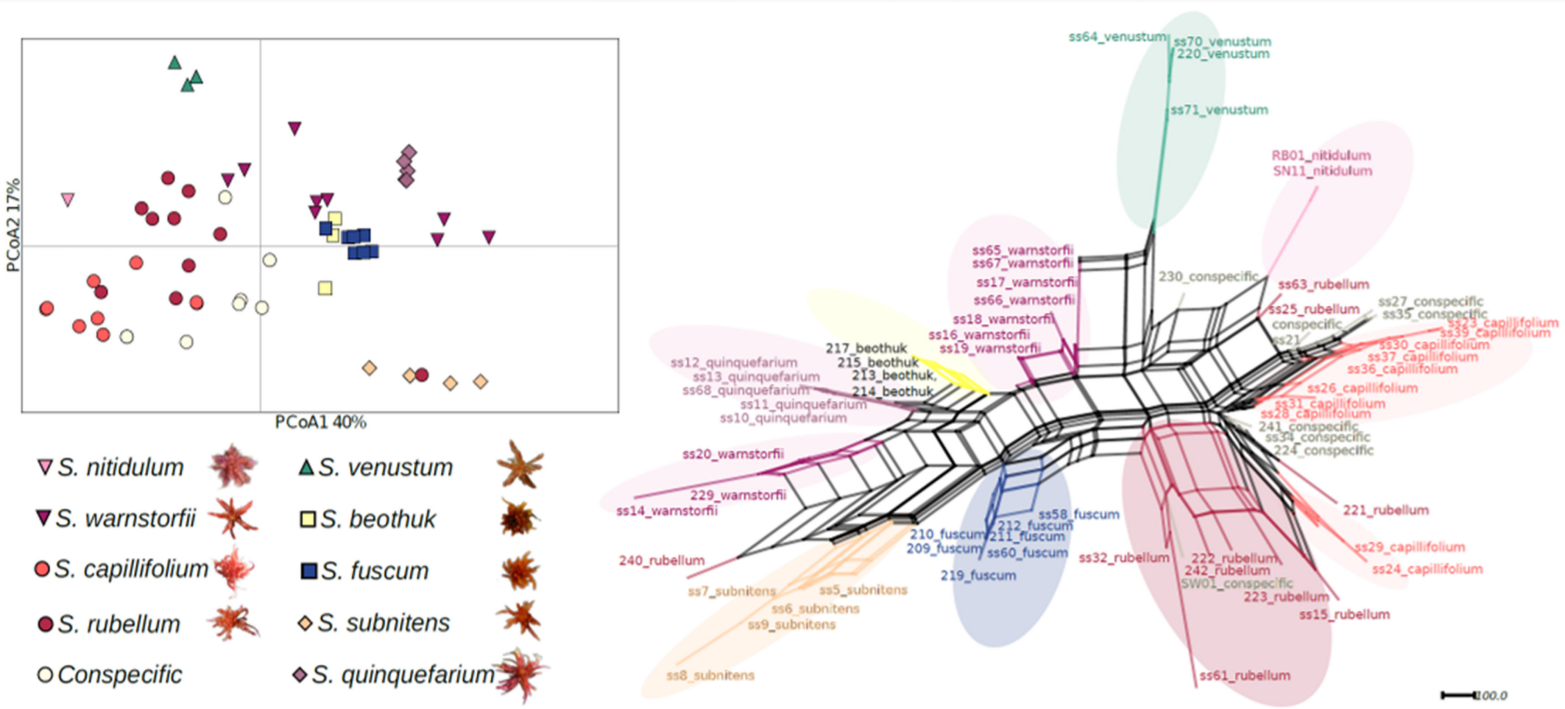
PCoA (left) generated in GenAlEx and Neighbor‐Net network (right) constructed in SplitsTree4, both based on genetic distance matrix calculated in GenAlEx6.501 of nine haploid *Sphagnum* species in subgenus *Acutifolia* and a group named “conspecific” that consists of specimens deviating morphologically and also somewhat genetically (see Structure results in Figure 3) from describe “red *Acutifolia*” species. Specimen ID in the network are the same as in Table 1, where voucher information is given.

The results of the PCoA show that there is large variation within *S. warnstorfii*, *S. rubellum*, *S. capillifolium*, and the conspecific specimens, and that they overlap to some extent (Figures 3 and 4). Also, one individual of *S. rubellum* group with *S. subnitens* in the PCoA, but not in the Structure analysis (results not shown). *Sphagnum venustum* is clearly separated from the rest, and *S. nitidulum* is close to, but genetically distant from the bigger group of “red *Acutifolia*” (Figure 4). The distinctness of *S. venustum* and *S. nitidulum* is also evident the Neighbor‐Net network generated in SplitsTree (Figure 4). However, in the PCoA analysis of the “red *Acutifolia*” dataset, *S. nitidulum* is found within specimens of *S. capillifolium* and *S. rubellum*. In the network, specimens of *S. subnitens*, *S. quinquefarium*, *S. beothuk*, and *S. fuscum* group in their respective species, while *S. warnstorfii*, *S. rubellum* and *S. capillifolium* are not fully resolved, with occurrences in several places in the network. Also, the conspecific specimens are spread among *S. rubellum* and *S capillifolium*.

## 4 DISCUSSION

Our findings seem to point to different paths leading to speciation in the subgenus *Acutifolia*. *Sphagnum skyense* is an example of instant speciation by polyploidization, a common speciation mode in *Sphagnum* (reviewed by Meleshko et al., 2018). We were able to identify the parents, which is not always possible (Kyrkjeeide et al., 2019; Kyrkjeeide, Hassel, Flatberg, Shaw, Yousefi, & Stenoien, 2016) as they may, for example, have gone extinct (Kyrkjeeide et al., 2019). Our genetical examination of *S. skyense* concludes that *S. subnitens* and *S. quinquefarium* are indeed the parental species. *Sphagnum subnitens* has two close relatives not included in the analyses: *S. subfulvum* Sjörs and *S. flavicomans* (Cardot.) Warnst. However, since they have been found to be genetically distinct based on microsatellite data (Kyrkjeeide et al., 2018), and *S. subnitens* and *S. skyense* mainly overlap in alleles, it is unlikely that either *S. subfulvum* or *S. flavicomans* are the parental species. Another allopolyploid species that has both parental species identified, is *S. troendelagicum* Flatberg (Såstad et al., 2001). Stenøien, Shaw, Stengrundet, and Flatberg (2011) estimated the origin of this species to about 40,000 years before present, indicating that the young age of the species make it possible to trace the parents. Both *S. troendelagicum* and *S. skyense* have narrow distribution ranges within areas glaciated during the last glacial maximum. Thus, *S. skyense* could have a recent origin like *S. troendelagicum*.

Genetic data show that *S. venustum* is a haploid species. Furthermore, it is genetically distinct from other similar *Acutifolia* species sharing the same habitat and geographical distribution range. Thus, we hypothesize that speciation may have taken place by niche differentiation along the mire structure gradient known in other *Sphagnum* species (Johnson et al., 2015).

Our PCoA result shows that *S. nitidulum* clusters within other ‘red *Acutifolia*’ species, but the haplotype is genetically distinct. We cannot conclude that it is a valid species based on our results, as a bigger sample size is needed to further explore whether it rather belongs to *S. rubellum*. *Sphagnum rubellum* is not with certainty collected from Terceira, and the included specimen collection of *S*. cf. *rubellum* shares its haplotype with *S. nitidulum*. The specimen of *S*. cf. *rubellum* from Terceira was collected in moist, sloping heath. As it is genetically identical to *S. nitidulum*, the genetic distinctness of the latter is likely not a result of adaptation to the sulfuric springs *S. nitidulum* is described from. A detailed morphological examination of the specimens was outside the scope of this study, but preliminary analysis indicate that the Azorean specimens collected as *S. nitidulum* and *S*. cf. *rubellum* are morphologically similar, further supporting no adaptation to an uncommon habitat. However, additional samples from Azores should be examined both morphologically and genetically to confirm which taxa occur at Terceira. *Sphagnum warnstorfii* is not reported from Terceira, but *S. capillifolium* is. It is necessary to study the phylogenetic relationship of the species to conclude on the validity of *S. nitidulum*, but also the origin of this taxon on Terceira. A larger selection of specimens from North America should be included in future analyses, as the Atlantic Ocean is a weak barrier to dispersal (Kyrkjeeide, Hassel, Flatberg, Shaw, Brochmann, & Stenoien, 2016; Stenøien et al., 2011).

The three red species, *S. warnstorfii*, *S. capillifolium*, and *S. rubellum*, form three distinct genetic groups, as expected (Shaw et al., 2005). However, the specimens collected as conspecific, group with *S. rubellum*, but overlap also with *S. capillifolium* and group close to *S. warnstorfii* in the PCoA. They have been identified as morphologically deviating, but conspecific to *S. warnstorfii* or *S. capillifolium*. Four specimens were collected as *S*. cf. *capillifolium* × *warnstorfii*, one was collected as *S*. cf. *warnstorfii*, and three more specimens were originally identified as *S. warnstorfii*, *S. capillifolium and S. rubellum*. A brief re‐examination of the morphology indicates that the conspecific group of specimens share characters and differ from other red *Acutifolia*, but a more thorough morphological examination and more genetic data should be included in a taxonomic revision to further explore this potential new taxon.

All three investigated taxa can be considered rare, since even though the amphi‐Atlantic *S. venustum* produces spores and has a wide distribution range, it seems to form very small populations where it occurs. It is only found at one site in Europe and assumed overlooked (Hallingbäck, 2019), and is assessed as data deficient in the European Red List of bryophytes (Hodgetts, Calix, et al., 2019). Both *S. skyense* and *S. nitidulum*, for the time being, are narrow endemics without known spore production, seemingly dependent upon fragmentation for dispersal. While *S. nitidulum* is evaluated in the IUCN global Red List of species as critically endangered (Gabriel & Sim‐Sim, 2019; Hodgetts, Calix, et al., 2019) due to the very small population size restricted to one site with declining habitat area, *S. skyense* occurs at several sites with no signs of decline and is thus evaluated as of least concern (Hodgetts, Calix, et al., 2019; Hodgetts, Lockhart, et al., 2019).

A morphological approach to describing species in the genus *Sphagnum* has proven useful, but due to large morphological variation along ecological gradients, like hight above water table, nutrient availability and light conditions, the determination of specimens can be very difficult both in the field and in the laboratory. Also, there is usually a time lag from the description of a new species until scientists and field biologist are reporting the species from new locations. *Sphagnum skyense* was described from Isle of Skye in 1988 (Flatberg, 1988a, 1988b), and then recorded again 16 years later. However, several new records were made the following years (Hill, 2014). All three species, but especially *S. venustum* and potentially *S. nitidulum*, should be searched for in mainland Europe. It could be that *S. venustum* has successfully established in Europe only once, but the species is small, with a somewhat dull color, and is therefore easy to overlook. The species is characterized morphologically (Flatberg, 2008) and included in floras (Flatberg, 2013; Laine et al., 2018), aiding field biologists with identification. On the other hand, if *S. nitidulum* is indeed a valid species, a morphological revision is needed to make it easier to distinguish it from other red *Acutifolia* species. In addition, a wider range of conspecific specimens should be investigated in terms of morphology, genetics, distribution ranges, and habitat preferences.

We have provided genetic data of three rare *Sphagnum* species with limited occurrences and assessments in Red lists. We have not studied intraspecific variation within these species, but found high genetic variation among the “red *Acutifolia*” species. Even though we were not able to fully resolve if *S. nitidulum* and the conspecific samples are genetically differentiated from *S. rubellum*, *S. capillifolium*, and *S. warnstorfii*, our findings indicate that there is genetic diversity within this group of species that should be prioritized for conservation. Ensuring protection of genetic variation safeguard biodiversity even in species groups where taxonomy is uncertain (Andrello et al., 2022; Rosauer et al., 2018). This is often the case for bryophyte species, where genetic diversity is not always expressed in identifiable morphological characters (Hedenäs, 2016), but is valid for all organisms groups were taxonomic work is limited due to high species diversity, few morphological characters or even lack of experts and taxonomists.

## AUTHOR CONTRIBUTIONS

**Magni Olsen Kyrkjeeide:** Conceptualization (equal); data curation (equal); formal analysis (lead); methodology (lead); visualization (lead); writing – original draft (lead); writing – review and editing (lead). **Olena Meleshko:** Conceptualization (equal); data curation (supporting); formal analysis (supporting); methodology (supporting); writing – review and editing (equal). **Kjell Ivar Flatberg:** Conceptualization (equal); data curation (equal); writing – review and editing (equal). **Kristian Hassel:** Conceptualization (equal); data curation (supporting); methodology (supporting); writing – original draft (supporting); writing – review and editing (equal).

## CONFLICT OF INTEREST STATEMENT

All authors declare that they have no conflicts of interest.

## Data Availability

The data are available in Table 2.
